# Some Past Incidents in the Life of the Dental Register

**Published:** 1900-01-15

**Authors:** 


### SOME PAST INCIDENTS IN THE LIFE OF THE DENTAL REGISTER.

BY THE EDITOR.

The DENTAL REGISTER OF THE WEST was born at the third annual meeting of the Mississippi Valley Association of Dental Surgeons, at Cincinnati, September 8, 1847. The following report from the proceedings of that meeting will tell the story: "The Committee on the Publication of a Dental Periodical reported that they had obtained statistics with respect to the cost of publishing a quarterly journal, and think it entirely within the ability of the society and the profession; we therefore advise the publication of such a periodical." Signed by Dr. James Taylor, Chairman of Committee. After considerable discussion, the society decided to publish a quarterly journal of at least forty-eight pages, the first issue to number 500 copies. Drs. S. P. Hullihen, B. B. Brown and James Taylor were elected publication committee.

The first number was issued in October, 1847, at Cincinnati, Dr. James Taylor doing the work of editor, also of publisher, as Dr. Hullihen declined to serve on the committee, and Dr. Brown, living in St. Louis, could give very little help on the initial number; he, however, materially assisted on the second number.

The first number contains the addresses delivered at the third annual meeting of the Mississippi Valley Dental Association, also the minutes.

One of the addresses was by Dr. A. Berry, on "Tobacco." It was characteristic of the man. It was a very learned composition, and closed with this admonition: "We should touch not, taste not the unclean thing. We should on all proper occasions give our testimony against the filthy, health-destroying habit of using tobacco in any form."

A very interesting communication, addressed to the editor by Professor Slack, says that he learns from perusing the New York *Dental Recorder* that a considerable controversy has been raised in New York by a local dental society in regard to a "quack," and a most reprehensible method of filling teeth with an amalgam of gold or silver, and he presumes more frequently with tin or lead. The writer presents quite an elaborate analysis of probable chemical and physical reactions which may take place when these materials are placed in the carious teeth. He concludes that as a principle, a pure metal, with but few exceptions, will be innoxious, but alloys easily attract oxygen and would be deleterious. The cellular tissues of the teeth, being filled with minute arteries, veins, absorbents and nerves, will absorb the deleterious oxides, which will be thrown into the circulation, and though the poison may move slowly at first, it will certainly, unless arrested, perform its work of destruction, and an early grave will be the portion of its victim.

The editor commenting on this communication, says he does not at that time know of more than two or three dentists of any note in the West who used amalgam or pretended to compare it favorably even with tin foil.

There are other articles in this number by such well-known men as Drs. John Allen, E. Taylor, H. R. Smith and M. Rogers. Dr. Sanford, of Chillicothe, Ohio, reports a case of third dentition, and Dr. Joseph Taylor, of Maysville, Ky., contributes a clinical report on the use of Dr. Morton's "Letheon"—ether. In this connection, Messrs. Stockton & Co. state that there is a probability that the English Government will make a large grant of money to Dr. Morton for his discovery, in which case he promises to refund all money he may have received for office rights.

The editor announces that all letters on business must have the postage prepaid, and he also expresses the hope that many physicians may subscribe for the REGISTER so that they may become intelligent on dental matters.

The volume is well edited, but the proof-reading is not so well done as we hope to do on the present volume. Taken as a whole it is a most interesting production for the time, and is very good reading even now.

In the second number, which appeared in January, 1848, the most interesting feature is a reprint of the account of the presentation to the Medico-Chirurgical Society of Edinburg, of chloroform as an anesthetic, by Dr. J. Y. Simpson, its discoverer, on the 10th day of November, 1847.

This number also contains a review of the proceedings of the American Society of Dental Surgeons, which held its meeting August 3, 1847, at Saratoga Springs, N. Y. The time of the meeting seems to have been almost entirely consumed in passing resolutions against using amalgam, and disqualifying such members as were using it and who refused to sign a pledge to quit it. The review is well written by Dr. E. Taylor, a brother of Dr. James Taylor, and abounds in wit and sarcasm. We wonder what sort of a report he would make of the meetings of some of the National organizations of to-day. The first volume contained many valuable contributions from the best men of the time, and much able writing by the editors.

In the first number of the second volume we find in the proceedings of the fourth annual meeting of the Mississippi Valley Dental Association the report of the Corresponding Secretary, who informs us that the REGISTER had last year a subscription list of eighty, but only seventy had paid. It also had five advertisers, three of whom had paid. The total receipts the first year were $155. (If this record is not broken this year we shall resign.) The editors' work was surely a labor of love; it was too well done to be paid for in dollars and cents. They were, however, voted unanimous thanks by the Association. In this number Dr. J. Taft makes his first appearance as a member of the Association, by contributing a paper on "The Use of the Key," which he condemns because of the principle on which it is used. Dr. Taft also contributes to the next number an article on the manufacture of an alcoholic lamp for laboratory and office uses. In fact, Dr. Taft takes a prominent part in the literature of the next few years, contributing to almost every issue something.

Dr. Berry contributes another of his characteristic letters, in which he humorously and sarcastically discredits "*Hill's Stopping*" and "*Odontalgic Drops*," although both are highly recommended by the mighty wise men of the East.

In the July, 1849, number is a communication from Dr. Thomas W. Evans, of Paris, France; in which he claims that he has an amalgam made of pure tin with which is combined a small quantity of cadmium, and which he says he has used with much success for a long time. By the way, in a subsequent issue he denies that he ever used it in his practice. He claims that it retains a good color, will not discolor the tooth, prevents recurrence of decay and is hard enough to resist the wear of mastication. For this contribution he gets a lot of criticism from all sides, as amalgams and their promoters were not popular at that date.

In this number also appears a bibliographical notice of the first edition of Harris' Dictionary of Dental Science.

This, the July, 1849, number, was delayed over a month in publication, because of an epidemic of cholera, which raged from May 10th to July 30th, carrying away by death 2,950 persons.

Financially the second year was not as successful a venture as the first. The receipts were less and the expenses greater. Dr. Taylor continued as editor, with Drs. Brown and Goddard as assistants.

Beginning with volume four in October, 1850, Dr. James Taylor was made sole editor and publisher, and the publication committee of three persons was dropped.

In the October, 1851, number is evidently the first effort made to report the discussion on papers read at the Association meetings. It is not a stenographic report, but reads well and seems to have been made by Dr. Leslie.

The January, 1853, number contains the report of the Congressional Committee, to which was referred the memorial of Dr. Wm. T. G. Morton, asking remuneration from Congress for the discovery of the anesthetic properties of sulphuric ether. The conclusion of the report, which considered all the testimony from Hippocrates down, was that to Dr. Jackson, the Boston chemist and claimant, belonged none of the credit for the discovery of the anesthetic virtues of ether, neither could Dr. Horace Wells claim any credit, as his experiments were confined entirely to investigation of nitrous oxide. The credit, therefore, falls to Dr. Morton.

This number of the REGISTER also contains a card by Dr. John Allen, announcing that he has brought suit against Dr. William Hunter for libel and infringement of his patents on a process for making continuous gum dentures. This was a famous controversy; old practitioners will recall the vigor with which it was conducted on both sides. In the July, 1855, number of the REGISTER the enterprise of the journal is shown by the publication in full the proceedings of the famous Allen vs. Hunter trial. The report was carefully made by an experienced reporter and revised by the editor. It occupied ninety pages, and included much valuable history and information on the method of making continuous gum dentures. No better description can now be found elsewhere.

In the October number the editor acknowledges the receipt of a specimen of crystal gold made by Drs. Taft and Watt, of Xenia, Ohio. In the January, 1854, number Dr. Taft gives a description of the gold and method of using it.

Much space was given about this time to the discussion of a method advocated for ventilating (?) the tooth pulp, suggested first by Dr. Hullihen, of Wheeling, W. Va., and called by him "*Risodontrophy.*" The discussion was a long one, and the treatment found favor with many of the best men in the country. Had they know the first principles of modern antiseptic surgery the practice would never have been countenanced, and many serious blunders would have been avoided.

At a meeting of the Ohio Dental College Association, February 21, 1854, Dr. John Allen resigned the chair of Operative Dentistry and Dr. J. Taft was elected to succed him. Dr. Taft held this chair for twenty-three years. At the same meeting the trustees were authorized to erect a building for college use at a cost not to exceed $5,000, which was done during the following year.

In the April, 1854, number, Dr. B. T. Whitney, of Buffalo, N. Y., contributes an interesting article on nitrate of silver, in which he advocates its use for obtunding sensitiveness of the teeth and for stopping decay. Thus antedating Dr. Stebbins by thirty-eight years.

With the close of volume nine Dr. Taylor, because of his large practice and the increasing burden of college duties, resigns as editor of the REGISTER, and is succeeded by Drs. J. Taft and George Watt.

Too much credit may not be given to Dr. Taylor for the successful manner in which he conducted the REGISTER through these nine years of its eventful history. He contributed largely of his own funds to support it financially, and gave lavishly of his time and mental resources to make it a faithful record of passing events. We should still more admire and honor him for the persistent and painstaking efforts he made to instruct the profession and elevate it to a high moral and scientific plane. To no other man is the high standard of our profession in the West more due than to Dr. Taylor.

We hope to continue this review of the REGISTER history in the next issue.

